# Nanocomposite-Enhanced Efficient Evaporation System for Solar-Driven Seawater Desalination—An Optimized Design for Clean Water Production

**DOI:** 10.3390/nano12193296

**Published:** 2022-09-22

**Authors:** Zhou Wei, Muhammad Sultan Irshad, Naila Arshad, Laila Noureen, Iftikhar Ahmed, Naveed Mushtaq, Muhammad Sohail Asghar, Qaisar Hayat, Uzma Ghazanfar, Muhammad Idrees, Naeem Shahzad, Yuzheng Lu

**Affiliations:** 1 Hubei Key Laboratory of Polymer Materials, Collaborative Innovation Center for Advanced Organic Chemical Materials Co-Constructed by the Province and Ministry, Ministry of Education Key Laboratory for the Green Preparation and Application of Functional Materials, School of Materials Science and Engineering, Hubei University, Wuhan 430062, China; 2 School of Environment and Energy, Peking University Shenzhen Graduate School, Shenzhen 518055, China; 3 ERC Research Centre, COMSATS University Islamabad, Lahore Campus, Islamabad 54000, Pakistan; 4 College of Physics and Optoelectronic Engineering, Shenzhen University, Shenzhen 518060, China; 5 Department of Physics, University of Wah, Wah Cantonment 47040, Pakistan; 6 Department of Physics, COMSATS University Islamabad, Islamabad 54000, Pakistan; 7 CE Wing, MCE, National University of Sciences and Technology Risalpur Campus, Risalpur 24090, Pakistan; 8 School of Electronic Engineering, Nanjing Xiaozhuang University, Nanjing 211171, China

**Keywords:** solar energy, water evaporation, anatase TiO_2_, AC, photothermal conversion, water scarcity

## Abstract

Solar-driven evaporation is a promising technology for desalinating seawater and wastewater without mechanical or electrical energy. The approaches to obtaining fresh water with higher evaporation efficiency are essential to address the water-scarcity issue in remote sensing areas. Herein, we report a highly efficient solar evaporator derived from the nanocomposite of anatase TiO_2_/activated carbon (TiO_2_/AC), which was coated on washable cotton fabric using the dip-dry technique for solar water evaporation. The ultra-black fabric offers enhanced solar absorption (93.03%), hydrophilic water transport, and an efficient evaporation rate of 1.65 kg/m^2^h under 1 kW m^−2^ or one sun solar intensity. More importantly, the sideways water channels and centralized thermal insulation of the designed TiO_2_/AC solar evaporator accumulated photothermal heat at the liquid and air interface along with an enhanced surface temperature of 40.98 °C under one sun. The fabricated solar evaporator desalinated seawater (3.5 wt%) without affecting the evaporation rates, and the collected condensed water met the standard of drinking water set by the World Health Organization (WHO). This approach eventually enabled the engineering design groups to develop the technology pathways as well as optimum conditions for low-cost, scalable, efficient, and sustainable solar-driven steam generators to cope with global water scarcity.

## 1. Introduction

The freshwater shortage has become a menace to humanity and the ecosystem due to growing population, industrialization, and urbanization, which have aggravated the crisis further due to the impact of global warming [1,2]. Although 97% of our plant is covered by water, most of it is unavailable due to freezing and salinity. Several technologies have been introduced to circumvent water scarcity by purifying the seawater, such as reverse osmosis (RO) and multistage flash (MSF) [3,4,5,6]. However, the installation of these technologies requires costly, sophisticated infrastructure, high energy consumption, and system maintenance. Solar energy is a promising renewable energy source that can meet all energy shortages across ecological and industrial boundaries as hourly incident solar radiation hitting the earth’s surface carries more energy than the annual global consumption [7,8]. Solar steam generation has recently gained extensive attention as a green energy resource to produce potable water from saline and wastewater to resolve the water shortage issue with the simultaneous preservation of the environment [9,10,11,12]. The efficiency of any steam generation device depends upon solar spectrum absorption capacity, hydrophilicity, thermal management, and salt rejection potential. Extensive efforts are invested in developing the photothermal conversion system for excellent solar harvesting, heat localization, and thermal management. Several photothermal materials are engineered (e.g., metallic nanoparticles, semiconductors, and carbon-based materials) to increase light absorption [6,13]. In fact, the nanomaterial plays a pivotal role in the capturing of enhanced solar flux followed by thermal conversion, whereas facile water availability and effective thermal insulation are two key features that require assistance from other elaborate designs. Various low-conductive hydrophilic substrates have been employed to provide facile water transportation and to achieve thermal management via their low thermal conductivity [8,14]. However, these solar steam generation (SSG) designs involve a complex fabrication process. Hence, some facile and efficient SSG systems are urgently required to accommodate all the essential features for the practical installment at the industrial level to meet the portable water scarcity issues.

In recent years, TiO_2_ has been explored in several applications because of its unique properties at the nano-level rather than bulk size [15,16]. The nanosized TiO_2_ owes its extremely large surface area to quantum confinement effects of charge-carriers and has been a useful element for the degradation of organic compounds in the wastewater, biocompatible inorganic photomedicine, bacterial effect, power generation, light detectors, photocatalysts, and solar cells owing to their high chemical stability and environmental friendliness [16,17,18,19,20]. Moreover, the anatase phase of TiO_2_ has gathered distinct attraction owing to its better photocatalytic efficiency over other polymorphs. At a small scale, in the TiO_2_ nanoparticles, the electron-hole recombination probability decreases, and hence more carriers are available for the oxidation or reduction process [21,22,23,24,25]. Furthermore, as one of the richest renewable biomass resources, activated carbon (AC) is low cost, non-toxic, and stability [2,26]. AC has been reported as good for photothermal conversion potential lying in its high surface-to-volume ratio owing to its small size, which facilitates the broadband solar absorption by abundant loosely bound π electrons held by weak Van Dar Waals forces [27]. Li et al. reported that the fabrication of activated carbon fiber for solar steam generation and achieved an evaporation rate of 1.22 kg m^−2^ h^−1^ with a photothermal conversion efficiency of 79.4% under 1 kWm^−2^ [26]. In various studies, the AC has also been modified by developing nanocomposites with different catalytic materials to optimize its relevant properties [2,27].

**Figure 1:**
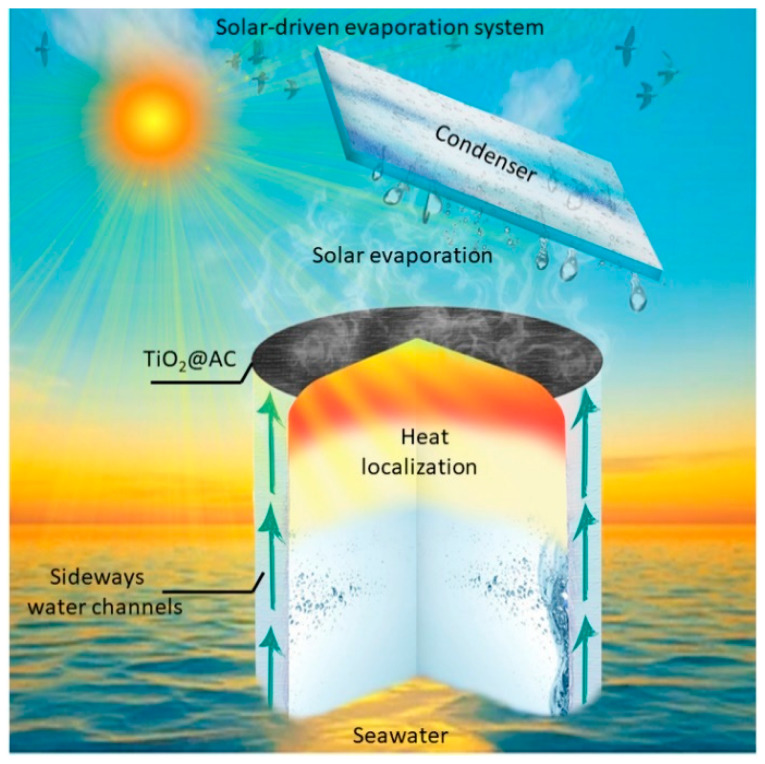
Schematic illustration of TiO_2_/AC-based solar evaporator, which endows efficient evaporation rates for effective desalination along with thermal management and self-floatability.

Herein, we report the development of a green, efficient, self-floating, super-hydrophilic, and TiO_2_/AC-based solar evaporator with a two-dimensional (2D) confined water path to suppress heat losses. The evaporation layer was composed of a TiO_2_/AC nanocomposite deposited cotton fabric (top surface). The hydrophilic fabric cloth was wrapped around the PET foam serving as a 2D water path for pumping water up to the evaporation structure. The polyethylene terephthalate (PET) foam was hydrophobic with a 0.3 Wm^−1^K^−1^ thermal conductivity and had a lower density than water [28]. The PET foam provided the device with the perfect thermal insulation and self-floating potential over wrapped cellulose fabric enabling the transportation of 2D water pumped by capillary force to the evaporation layer, as illustrated in Figure 1. These sideways water channels and centralized thermal insulation design allow accumulating photothermal heat at the liquid and air interface, maintaining a surface temperature of 40.98 °C under one sun without affecting the evaporation rate. As PET can float over water, only the bottom side of the cotton fabric is in direct contact with saline water. Although many methodologies have previously provided ample thermal insulation, only this work has presented and elaborated the enhanced solar absorption in the order of 93.03% with a facile water pathway at a higher evaporation rate of 1.65 kg/m^2^h under 1 kW m^−2^ or one sun irradiation. Therefore, this study offered fundamental design technology pathways as well as optimum conditions for the development of scalable, efficient, and sustainable solar-driven steam generators to cope with the global water scarcity dilemma.

## 2. Materials and Methods

### 2.1. Materials

The pellets of activated carbon were bought from Wuhan BASF Chemical Industries Industry Co., Ltd. Wuhan, P.R. China. The titanium isopropoxide, acetylacetone, and absolute ethanol were bought from Aladdin Industrial Corporation (Shanghai, China). While the polyethylene terephthalate (PET) foam and cotton fabric were offered by Sinopharm Chemical Reagent Co., Ltd., Beijing, China. All the purchased chemicals were met the 99% purity level and were employed for material fabrication without any further purification process.

### 2.2. Synthesis of TiO_2_ Nanoparticles

First, the TiO_2_ was synthesized by preparing four different solutions and labeled as A, B, C, and D with the following concentrations: (1) 40 mL of deionized (DI) water beaker A (2) 40 mL absolute ethanol in a beaker B; (3) 0.5 g of urea dissolved in 10 mL of DI water in beaker C; (4) 2 mL acetylacetone (first) and 2 mL titanium (second) isopropoxide in a 10 mL measuring cylinder D. The solution B was continuously stirred and then solution D was mixed in B under continuous stirring at room temperature. Afterward, the resulting solution and solution C were added drop-by-drop in beaker A at room temperature while constantly stirring. A pale-yellow solution was obtained and measured with a pH value up to 5.6, showing an acidic nature, which was favorable for the TiO_2_ preparation. The resulting acid was stirred for another hour and then transferred into a 120 mL Teflon-lined stainless-steel autoclave and heated in an oven for 20 h at 150 °C and for 18 h for the completion of the reaction. Then, the autoclave was removed from the oven and allowed to freely cool at room temperature. The obtained product was yellowish-white, which was then washed and centrifuged with DI water and absolute ethanol several times until all the undesired impurities were eliminated. Finally, the sample was dried at 85 °C for 4 h. The final product was collected and ground in a mortar pestle for 5 min to obtain fine powder, which was further modified.

### 2.3. Synthesis of TiO_2_/AC Nanocomposites

The obtained activated carbon pellets were transformed into fine powder by grinding using an electronic crushing machine and ball milling for three hours. The collected AC powder was further grinded using a mortar pestle to obtain a fine powder. Then, the TiO_2_/AC nanocomposite was prepared by a solid-state reaction method. For this, 3 g of AC was mixed into 1 g of TiO_2_ and ground in a mortar pestle to form a homogeneous powder.

### 2.4. Fabrication of TiO_2_/AC Solar Evaporator

The fabrication of the TiO_2_/AC solar evaporator was incorporated via the dip-dry technique. For this, the calculated amount (2 g) of as-prepared TiO_2_/AC powder was dissolved into 100 mLDI water and sonicated to form the homogeneous solution. The cotton fabric was cut into 2 × 2 cm^2^ dimensions and put in a petty dish. Afterward, the prepared homogeneous mixture was dropped onto the cotton fabric with a dropper and dried in the oven at 60 °C. When the water completely evaporated from the cotton fabric, the TiO_2_/AC nanocomposite was embedded inside the fabric giving it a light grey color. The sonicated solution was again dropped and dried over several cycles until the color of the cotton fabric turned pitch black. Then the PET foam was cut into 2 × 2 cm^2^ dimensions and wrapped with the two extended sides of cotton fabric to bestow floatable and thermal insulation on the device. The prepared photothermal layer was then examined for solar steam generation testing.

### 2.5. Solar Evaporation Setup

The solar-driven evaporation experiments were conducted via a solar simulator (PLS-FX300HU, Beijing Perfect light Technology Co., Ltd., Beijing, China) capable of simulating multiple solar intensities. An optical filter was employed to provide a 1.5 G AM spectrum. The as-prepared TiO_2_/AC was floated over the water surface in a petty dish for the steam generation experiments. The solar intensity was set at 1 kW m^−2^ (one sun), and the device was placed under a solar beam spot. The time-dependent mass change was recorded using an electronic analytical balance (Mettler Toledo, ME204, Singapore ) with a resolution of 0.001 g. The whole setup was allowed to stabilize for 30 min, and the evaporation rate of the system was measured under one sun illumination. The surface temperature was measured using a thermal infrared image camera (FLIR E4 Pro, Wuhan Guide Sensmart Tech Co., Ltd., Wuhan, China) which employed two temperature sensing thermo-couples mounted on the photothermal surface and bottom surface, respectively. An inductively coupled plasma-optical emission spectrometry (CP-OES, EP Optimal 8000, Perkin Elmer, San Jose, CA, USA) was employed to measure the salt ion concentrations in the saline water and condensed water. The whole experimental process was conducted under ambient conditions, at a temperature (~26 °C) and humidity of ~46%.

### 2.6. Photothermal Conversion Efficiency

The photothermal conversion efficiency of the developed systems was calculated by the following equations [5]:

(1) η=ṁhLVqi

(2) $$h_{LV}=\lambda +C\Delta T$$

where *ṁ* denotes the evaporation rate under simulated solar irradiation intensity subtracting the evaporation rate of simple bulk water (the mass flux) and evaporation rate in the absence of light, *h_LV_* is the liquid-vapor phase change enthalpy with sensible heat, *q_i_* represents the simulated solar intensity (1 kW m^−2^), *λ* denotes the latent heat phase change (latent heat changes from 2430 kJ kg^−1^ at 30 °C to 2256 kJ kg^−1^ at 100 °C), C gives the specific heat capacity of water (4.2 kJ kg^−1^K^−1^), and Δ*T* shows the enhancement in water temperature [5]. The whole experiment was performed at ambient environmental conditions, i.e., temperature (26 °C) and humidity (46%).

## 3. Results

### 3.1. Structural Analysis

**Figure 2:**
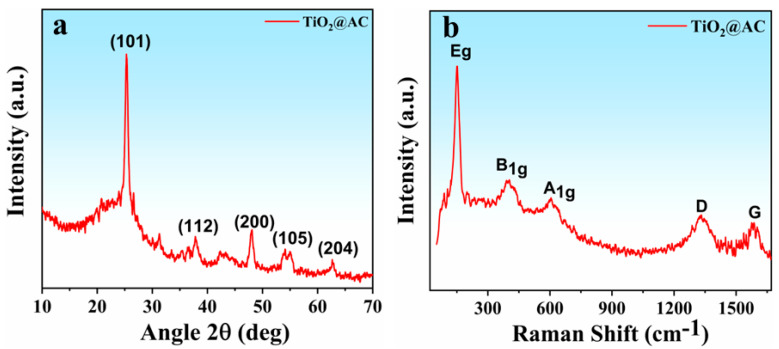
(**a**) XRD spectra of TiO_2_/AC nanocomposites. (**b**) Raman spectra of TiO_2_/AC nanocomposites.

The X-ray diffraction analysis was performed to assess the crystalline nature and phase composition of the as-prepared TiO_2_/AC nanocomposite. The obtained XRD pattern of the resulting material in the range of 2θ = 10–70° is shown in Figure 2a, revealing the crystalline tetragonal structure of TiO_2_. All the Bragg diffraction peaks are positioned at the 25.27, 37.83, 48.12, 54.12, and 62.71° angles with the corresponding index planes (101), (112), (200), (105), and (204), respectively, in a perfect match with anatase phase TiO_2_ with tetragonal arrangement [29]. Srinivasu et al. [29] reported the same XRD pattern for the anatase titania. Furthermore, the structural information TiO_2_/AC was also affirmed by the Raman spectroscopy, and obtained spectrum is shown in Figure 2b. The Raman spectra revealed the three characteristic bands of anatase TiO_2_ with symmetries e.g., B1g, and A1g appearing at 152.42, 37.70, and 605.73 cm^−1^, respectively [30]. Whereas the last two peaks, appearing from 3000–500 cm^−1^, corresponded to the D and G bands of activated carbon. The peak at 1331.04 cm^−1^ corresponded to the D band, which appeared from the sp^2^ hybridization, while the other, at 184.60 cm^−1^, fell in the G band, which arose from the stretching of the C-C bonds and determined the physical properties [31].

### 3.2. Morphological Analysis

**Figure 3:**
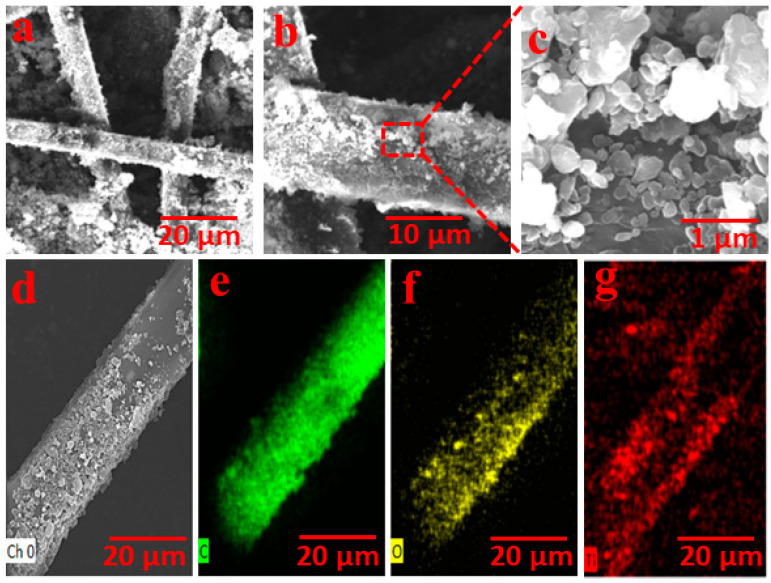
The structural and morphological investigations of TiO_2_/AC-based solar evaporator. FESEM images of the (**a**) TiO_2_/AC deposited cotton surface showing rugged surface texture intensified pitch-black color on hydrophilic nodes of cotton fabric. (**b**) Homogenized coating on TiO_2_/AC on a single thread. (**c**) FESEM image of TiO_2_/AC nanocomposite embedded on the surface of cotton fabric. (**d**–**g**) EDS mapping of TiO_2_/AC embedded cotton fabric.

The highly efficient TiO_2_/AC-based solar evaporator was developed via a facile fabricating technique. The hydrophilic cotton fabric was etched on the top surface by TiO_2_/AC photothermal materials to enhance the diffuse reflection via the facile dip-dry technique. Both sides of the hydrophilic fabric cloth were wrapped around the PET foam for 2D water transportation or convective sideways water channels. The PET foam was a good thermal insulator (0.3 Wm^−1^K^−1^) with hydrophobicity and is floatable on the water surface. Moreover, 2D water transportation reduced heat losses due to the confined dimensionality. The morphological and microstructural analyses were assessed by FESEM microscopy. Figure 3a shows the FESEM image TiO_2_/AC loaded cotton fabric revealed the uniform deposition over the surface with a dense-rough texture. Figure 3b shows the high-resolution image of a single cotton fabric thread over which TiO_2_/AC is evenly distributed. Figure 3c reveals the morphology of TiO_2_/AC composite at a 1 µm scale imprinted on the surface of a single thread. The highly absorbing TiO_2_/AC composite imparted the surface with excellent solar flux capturing the potential and formed an inset of dark surface structure, which enhanced the surface absorption and improved the light absorption potential due to its intrinsic diffuse reflection specificity. Furthermore, the elemental dispersion spectrum (EDS) was conducted to survey the elemental composition of TiO_2_/AC loaded cotton fabric. The mapping of the top photothermal layer confirmed the presence of C, O, and Ti, as shown in Figure 3d–g, respectively.

### 3.3. Solar Absorption and Interfacial Surface Temperature

**Figure 4:**
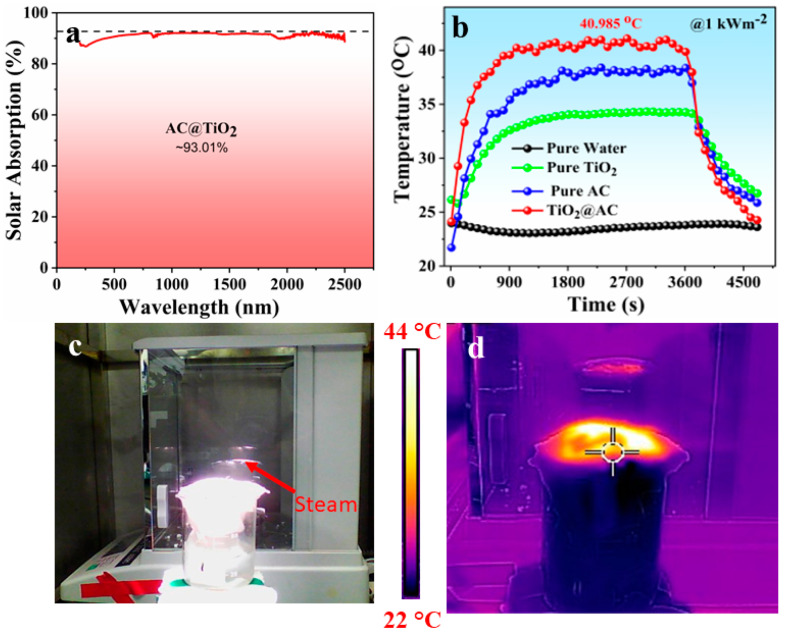
(**a**) UV-Vis’s spectra of TiO_2_/AC nanocomposite showing supreme solar absorption. (**b**) The surface temperature changes of the comparative four systems, i.e., pure water, pure TiO_2_, pure AC, and TiO_2_/AC under one sun. (**c**,**d**) Real-time demonstration of vapor generation and respective IR image of TiO_2_/AC solar evaporator under one sun irradiation.

The solar absorption of the as-prepared TiO_2_/AC was determined by UV-Vis’s spectroscopy which is provided by an integrated sphere through the whole solar spectral length (200–2500 nm), and the obtained spectrum is shown in Figure 4a. The composite material showed an excellent solar absorption (93.01%) over the whole spectral range which can be attributed to the addition of AC. The facile deposition of TiO_2_/AC over cotton fabric bestowed a rough structure to the top photothermal layer and enabled the diffuse reflection of incident light, and ultimately a high surface temperature was achieved. Figure 4b shows a comparative analysis of surface temperature enhanced under one solar intensity for the developed four systems, i.e., pure water, pure TiO_2_, pure AC, and TiO_2_/AC-based solar evaporator. As seen, the temperature of the TiO_2_/AC surface rises rapidly to 35.3 °C within 6 min as compared to water and TiO_2_ under the same solar irradiation showing the rapid response of the photothermal transition process. A maximum surface temperature elevation of up to 40.9 °C was achieved by the TiO_2_/AC system under 1 kW m^−2,^ which is higher than pure water, pure TiO_2_, and pure AC.

Figure 4c shows the digital image of the TiO_2_/AC-based device demonstrating the real-time solar evaporation under one solar intensity. The corresponding IR image is shown in Figure 4d, manifesting the heat aggregation within an insulating structure, i.e., excellent thermal localization on the top surface with no heat conduction to the downward structure. Hence, the above results prove that as prepared TiO_2_/AC device is enabled by excellent photothermal with simultaneous thermal insulation of downward matrix.

### 3.4. Solar Evaporation Performance

**Figure 5:**
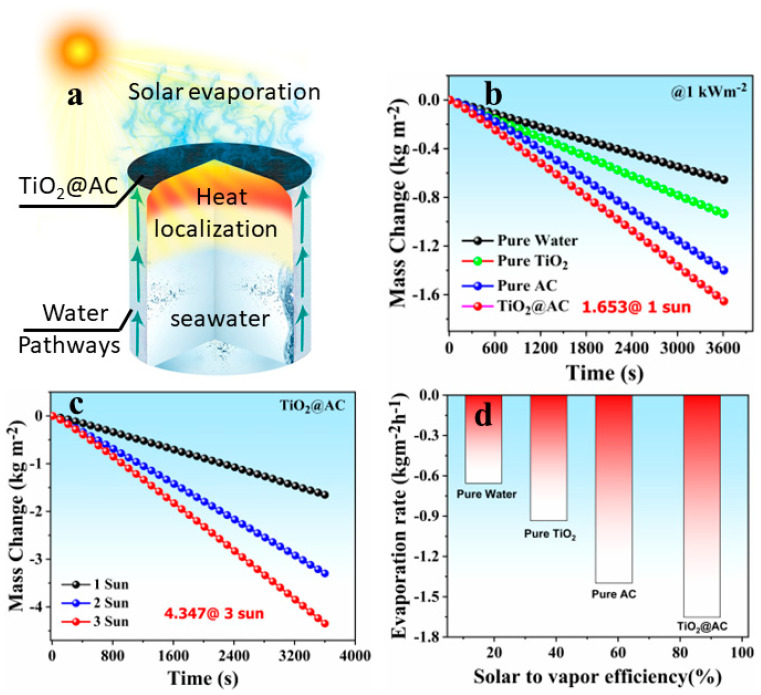
(**a**) Schematic illustration of the highly efficient TiO_2_/AC based solar evaporator showing excellent thermal sustainment on the top surface and of sideways water channels of cotton fabric facilitated smooth water transport of water while centralized PET suppressed heat losses and self-floating potential. (**b**) Comparative time-dependent mass change of pure water, TiO_2_, AC, and TiO_2_/AC solar evaporator under one sun irradiance. (**c**) Mass change of TiO_2_/AC solar evaporator for different solar intensities up to 3 kW m^−2^. (**d**) Comparative evaporation rates and solar-to-vapor conversion efficiencies of pure water, TiO_2_, AC, and TiO_2_/AC solar evaporator evaporating under one sun.

The top surface of the cotton fabric was deposited by the high-absorption TiO_2_/AC-based photothermal material for the direct harnessing of the incident solar light. The other sides of the fabric are wrapped around the PET foam, which acted as excellent thermal insulation and enabled the device to self-float over the water surface. As schematically illustrated in Figure 5a, the localized heat was perfectly accumulated on the top surface, while the downward conduction was restricted via the low conductivity of PET foam. The cotton fabric wrapped around PET enabled the 2D water transportation pumped by capillary force to the evaporation layer. As PET can float over water, only the bottom side of the cotton fabric is in direct contact with bulk water. Compared with the “3D bulk-water supply”, the heat losses through the 2D were reduced due to the reduced dimensionality. Therefore, with a 2D water-surface path, systematic water transport with suppressed parasitic heat losses was simultaneously achieved. The anchoring of photothermal material on the photothermal layer bestows rugged and dense surface texture with the supreme absorbing potential of TiO_2_/AC enabling an increased photothermal conversion rate and ultimately a high evaporation rate.

Herein, we conducted a comparative analysis of the photothermal conversion process, i.e., pure water, pure TiO_2_, and TiO_2_/AC. Figure 5b shows the time-dependent mass change of developed systems under continuous solar irradiation (1 kW m^−2^) for 1 h by a weighing balance with 0.0001 mg precision. The TiO_2_/AC was recorded with a maximum mass change up to 1.653 kg m^−2^. Furthermore, the time-dependent mass change for TiO_2_/AC was also recorded under multiple solar intensities, and the obtained data are shown in Figure 5c. The maximum mass change obtained by the TiO_2_/AC is up to 4.347 kg m^−2^ under three solar illuminations (3 kW m^−2^). Moreover, the corresponding evaporation rates and solar to vapor efficiency were also calculated for the respective four systems and obtained data are shown in Figure 5d. The TiO_2_/AC achieved the highest evaporation rate, 1.653 kg m^−2^ h^−1^, and its optimized solar to vapor efficiency, 87.1%, was significantly higher than pure TiO_2_, pure AC, and many other steam generating systems reported previously.

### 3.5. Purity and Reliability

**Figure 6:**
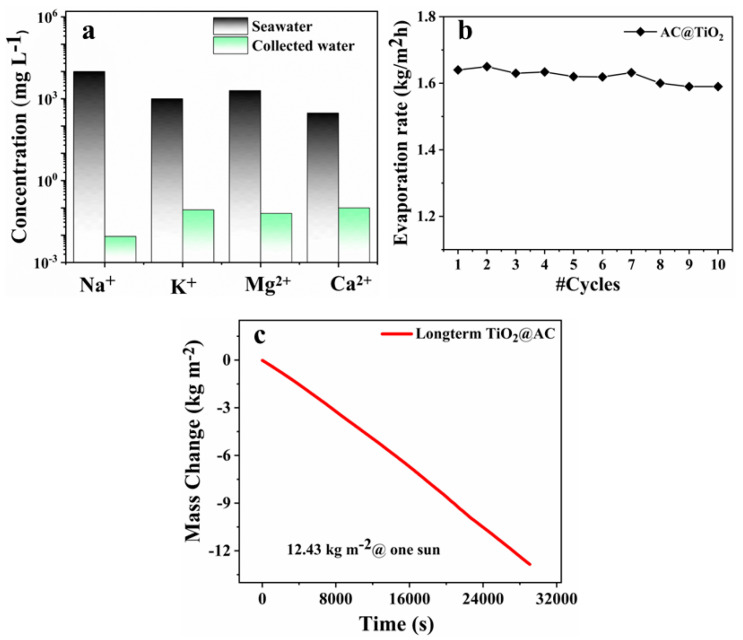
Self-regenerating and salt-resistant performance of in-situ polymerized ISPN solar evaporator. (**a**) Inductively coupled plasma-optical emission spectrometry (ICP-OES) examination of a concentration gradient of primary salt ions in stimulated seawater and condensed water. (**b**) The number of washing cycles vs. evaporation rates of TiO_2_/AC solar evaporator. (**c**) Long-term evaporation performance under one sun solar irradiance.

The TiO_2_/AC solar evaporator has a high potential to efficiently purify saline water. The seawater contains high concentrations of primary salt ions, which are essential to reject to obtain standard drinking water. For this, we employed the Inductive Coupled Plasma Atomic Emission Spectroscopy (ICP-OES) to investigate the concentration percentage of salt ions in stimulated water (3.5 wt%, NaCl) and the condensed water obtained from the TiO_2_/AC solar evaporator. A comparative analysis was made of the primary salt ions concentration, i.e., Na^+^, K^+^, Ca^2+^, and Mg^2+^, before and after desalination, and the obtained data are shown in Figure 6a. As is obvious from the graphs, a significant drop in the ion’s concentration was achieved in the condensed water as compared with the ions’ concentration in simulated seawater.

The water purified by the TiO_2_/AC solar evaporator perfectly rejected the elevated ions’ concentration level, and the quality of the produced fresh water perfectly met the standards for drinking water set by the World Health Organization (WHO). In most steam-generation devices, a serious problem is the surface degradation of the photothermal layer when treated continuously over several cycles, which tends to decrease the evaporation rate of the device and lowers its efficiency. The stability and surface degradation of a device is of key importance for its practical applicability and reliability. To check the stability and anti-foiling nature of TiO_2_/AC solar evaporator, it was continuously operated over several cycles (10 cycles) to check the consistency in evaporation rates. As shown in Figure 6b, smooth evaporation rates were obtained with a negligible discrepancy seen in the evaporation showing the excellent reliability of our device. Moreover, long-term evaporation performance revealed that the TiO_2_/AC solar evaporator produced efficient freshwater generation (12.43 kg.m^−2^) during consecutive 8 hr evaporation under one sun solar irradiance without any surface degradation, as illustrated in Figure 6c. Indeed, there is ample improvement required in highly efficient solar evaporation structures for high throughput freshwater generation in all weather conditions to minimize the infant stage between the status quo and real-world applications. These improvements are low-cost, flexible vapor condensers and have limitations due to the intermittent nature of sunlight. Hence, the prepared device can potentially be installed at the industrial level for real-world applications.

## 4. Conclusions

The report can be summarized as the fabrication of a highly efficient solar evaporator based on the anatase TiO_2_/activated carbon (TiO_2_/AC) nanocomposite, which was anchored on the super-hydrophilic cotton fabric via the facile dip-dry technique. The pitch-dark coating of TiO_2_/AC nanocomposite on cotton fabric endorsed its rugged surface texture and facilitated enhanced solar absorption (93.03%), facile water transportation, and a high evaporation rate of 1.65 kg/m^2^h under 1 kW m^−2^, or one sun irradiation. More importantly, the sideways water channels and centralized thermal insulation of the designed TiO_2_/AC solar evaporator accumulated photothermal heat at the liquid and air interface along with an enhanced surface temperature of 40.98 °C under one sun. The fabricated solar evaporator desalinated seawater (3.5 wt%) without affecting the evaporation rate, and the collected condensed water met the standards for drinking water set by the World Health Organization (WHO). This approach continued to produce freshwater using low-cost and less toxic solar evaporators and has the potential to address the severe water shortage in a more sustainable way in the world.

## Data Availability

Not applicable.
